# Supplementary material for: Experimental warming and drying increase older carbon contributions to soil respiration in lowland tropical forests
Source: Nat Commun. 2024 Aug 17;15:7084. doi: 10.1038/s41467-024-51422-6 (PMC11330460; doi:10.1038/s41467-024-51422-6)
Supplement: Supplementary file 1 — Supplementary Information [file 41467_2024_51422_MOESM1_ESM.pdf]

Experimental Warming and Drying Increase Older Carbon Contributions to Soil Respiration in Lowland Tropical Forests

SUPPLEMENTARY INFORMATION

Supplementary Figures 1–8

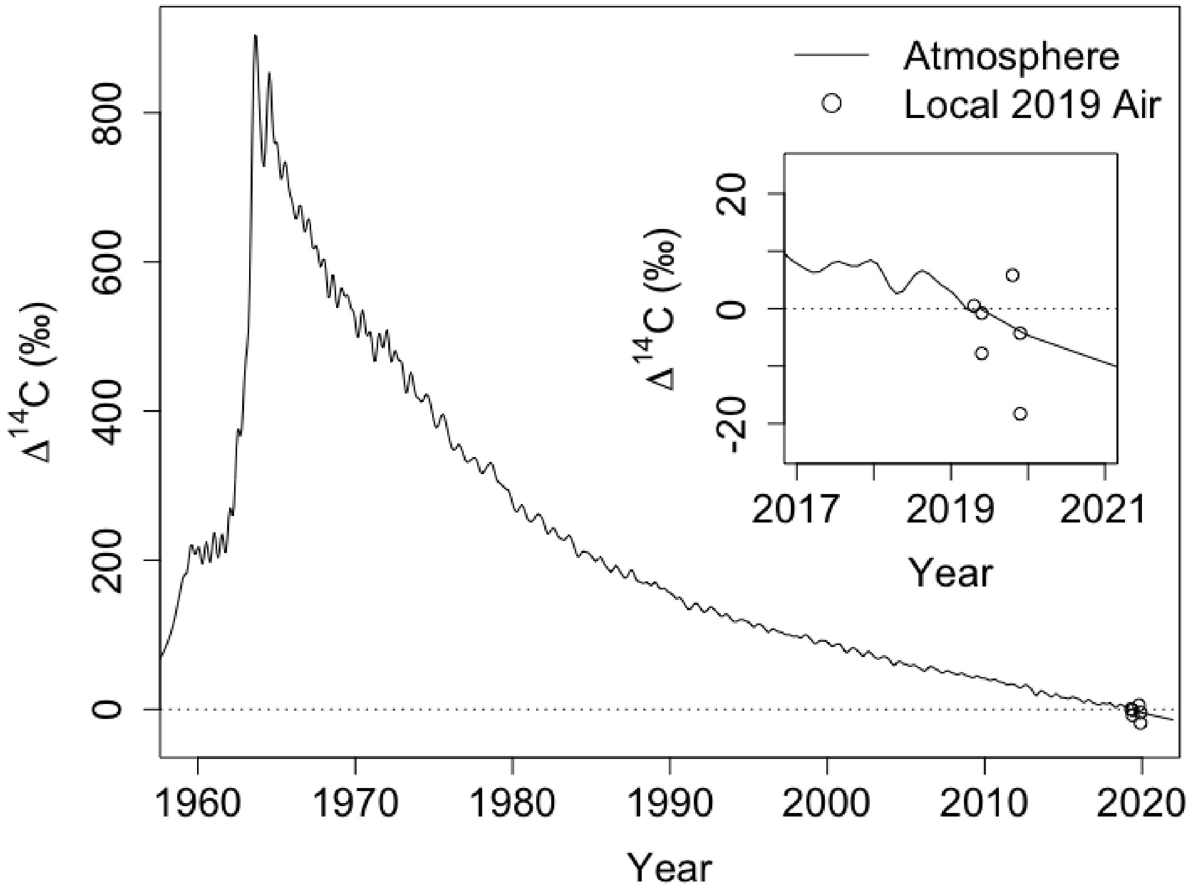

Supplementary Fig. 1. Northern Hemisphere Zone 2 atmospheric Δ^{14}C values (black line)[1] and Δ^{14}C value of air samples collected from the sites in this study in 2019 (circles). Values for 2019 to 2022 are extrapolated using a linear regression fit to data from 1995 to 2019, which yields an atmospheric decline of 4.6 ± 0.2 permil per year and an R^2 of 0.99. The mean Δ^{14}C value of air samples collected from the sites in this study in 2019 was -4 ± 3 ‰ (± standard error, n = 6).

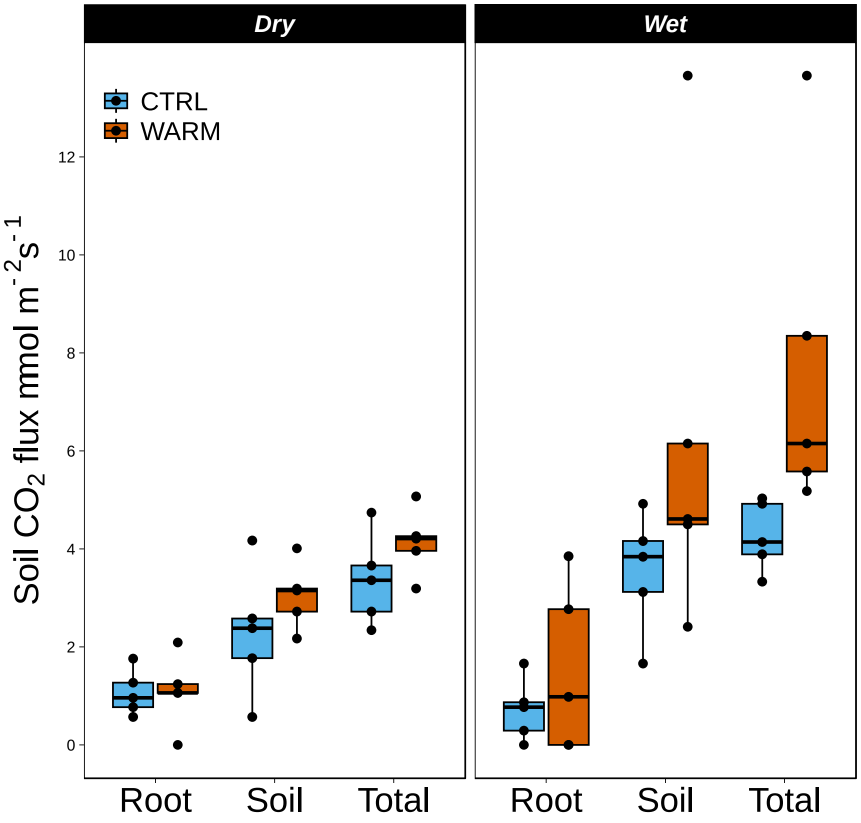

Supplementary Fig. 2: Total soil CO_2 flux rate at SWELTR partitioned into the root-derived (Root) and soil heterotroph-derived (Soil) components (see Methods). Flux rates are monthly averages for March (dry season) and October (wet season) in 2019 (n = 5 plots). Total flux rates are the same as those shown in Fig. 1a of the main text and are shown here for ease of reference. The figures show plots warmed by +4ºC (WARM) and controls (CTRL). Lines indicate medians, ends of boxes show the upper (Q3) and lower (Q1) quartiles, whiskers indicate minimum and maximum ranges (calculated from quartiles), solid points are individual observations. This partitioning was not affected by experimental warming ($p = 0.75$) or season ($p = 0.35$) as tested by two-way ANOVA with treatment and season (Supplementary Table 4).

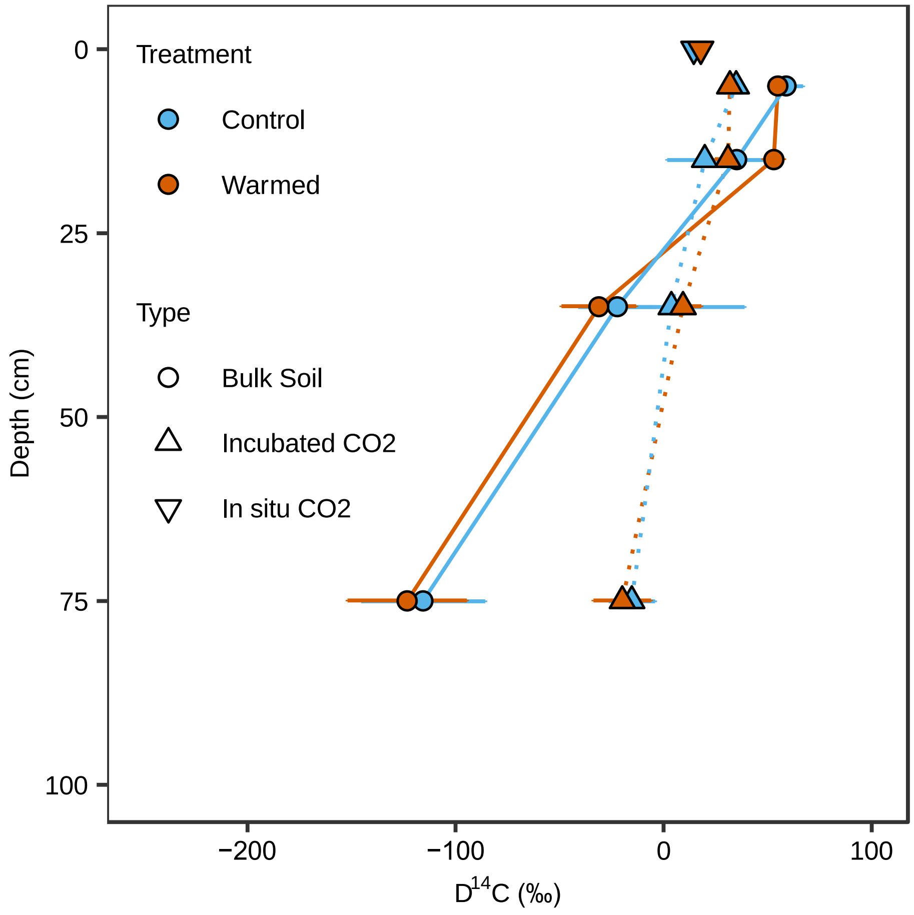

Supplementary Fig. 3: Δ14C values for bulk soil, CO_2 from laboratory soil incubations, and in situ surface soil CO_2 efflux at SWELTR. In situ soil CO_2 efflux Δ14C values are the same as those shown in Fig. 4 and are replotted (for all collar types and seasons) for reference. Bulk soils were collected from all plots in October 2019 from the following depth increments: 0–10, 10–20, 20–50, and 50–100 cm. The figure shows means as large symbols with standard errors and individual measurements as small symbols. n = 5 for bulk soils and for incubations of 0–10 cm depth. n = 3 for incubations below 10 cm depth. Δ14C values of CO_2 from incubations in the top 20 cm were higher than in situ CO_2 ($p < 0.01$) as tested with a two-way ANOVA with C source and treatment. Effects of experimental warming and differences between Δ14C values for bulk soils and CO_2 from incubations were tested using a three-way repeated measures ANOVA with C source, treatment. This test indicated a significant interaction between C source and depth ($p < 0.01$) and comparisons of slopes with depth indicated that bulk soil Δ14C values declined more with depth than CO_2 from incubations ($p < 0.01$).

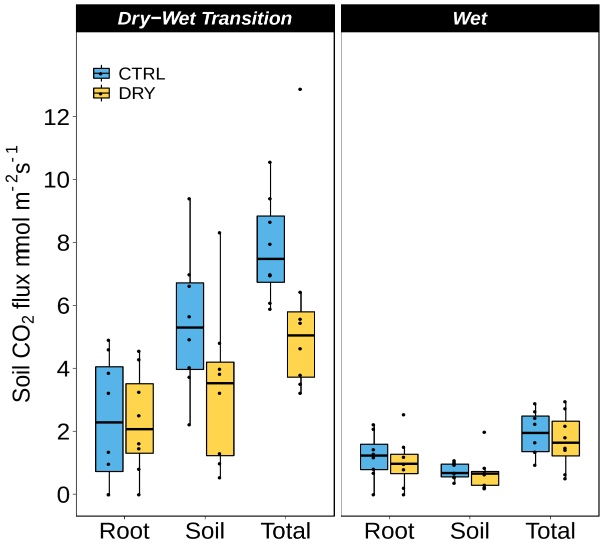

Supplementary Fig. 4: Total soil CO_2 flux rate at PARCHED partitioned into the root-derived (Root) and soil heterotroph-derived (Soil) components. Flux rates are single time-point measurements for May (dry-to-wet season transition) and November or December (wet season) in 2019 (n = 5 plots). Total flux rates are the same as those shown in Fig.2a of the main text and are shown here for ease of reference. The figures show plots with 50% of throughfall excluded (DRY) and controls (CTRL). Lines indicate medians, ends of boxes show the upper (Q3) and lower (Q1) quartiles, whiskers indicate minimum and maximum ranges (calculated from quartiles), solid points are individual observations. See Supplementary Fig. 5 for sites plotted individually and statistical test results.

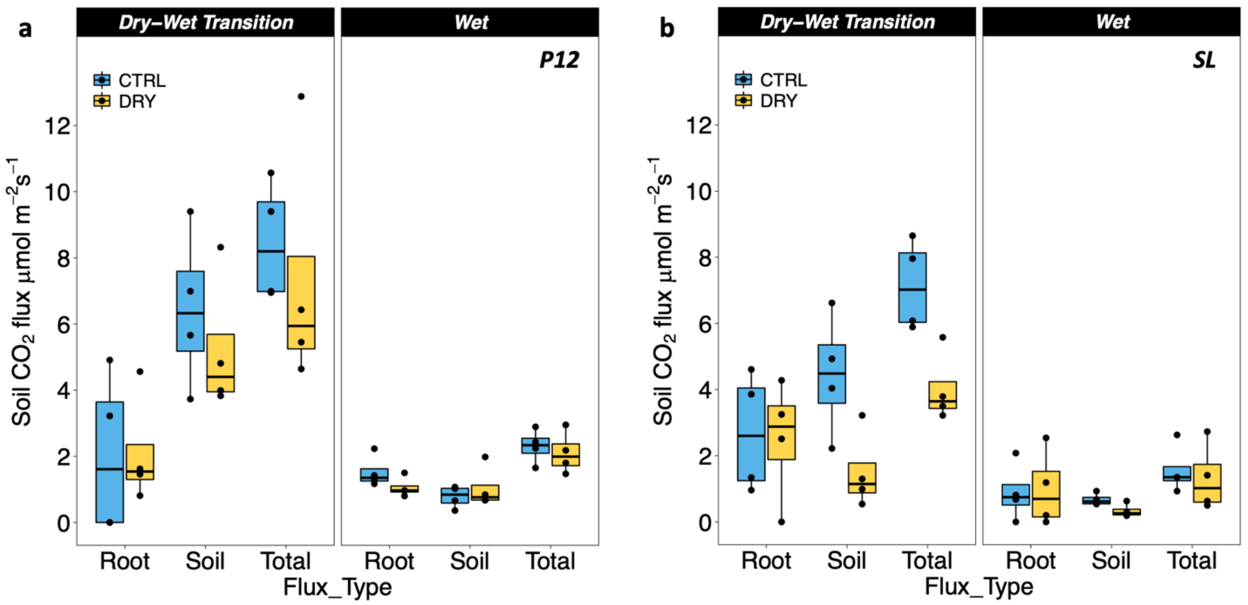

Supplementary Fig. 5: Total soil CO_2 flux rate at P12 (a) and SL (b) partitioned into the root-derived (Root) and soil heterotroph-derived (Soil) components. Flux rates are single time-point measurements for May (dry-to-wet season transition) and November or December (wet season) in 2019 (n = 5 plots). Total flux rates are the same as those shown in Fig. 4 of the main text and are shown here for ease of reference. The figures show plots with 50% of throughfall excluded (DRY) and controls (CTRL). Lines indicate medians, ends of boxes show the upper (Q3) and lower (Q1) quartiles, whiskers indicate minimum and maximum ranges (calculated from quartiles), solid points are individual observations. Of total soil respiration, 58 ± 6 % at P12 and 51 ± 8 % at SL was heterotrophic. Effects of throughfall exclusion and season on total, heterotrophic, and root CO_2 efflux and the partitioning of total CO_2 efflux were tested using a three-way repeated measures ANOVA with site, treatment, and season (Supplementary Table 6–8). Heterotrophic CO_2 fluxes were higher at the drier site (P12) than the wetter site (SL) during the dry-to-wet transition ($p < 0.01$, Supplementary Table 7). This partitioning was not affected by throughfall exclusion and did not differ significantly between sites (Supplementary Table 9), but a significant interaction of site and season ($p = 0.02$) indicated that at P12, more of the total soil CO_2 efflux was attributed to heterotrophs during the dry-to-wet season (75 ± 6 %) than in the wet season (41 ± 5 %). This pronounced increase in heterotrophic respiration during the dry-to-wet transition at P12 was likely driven by larger rewetting effects at the drier site, compared to the wetter site.

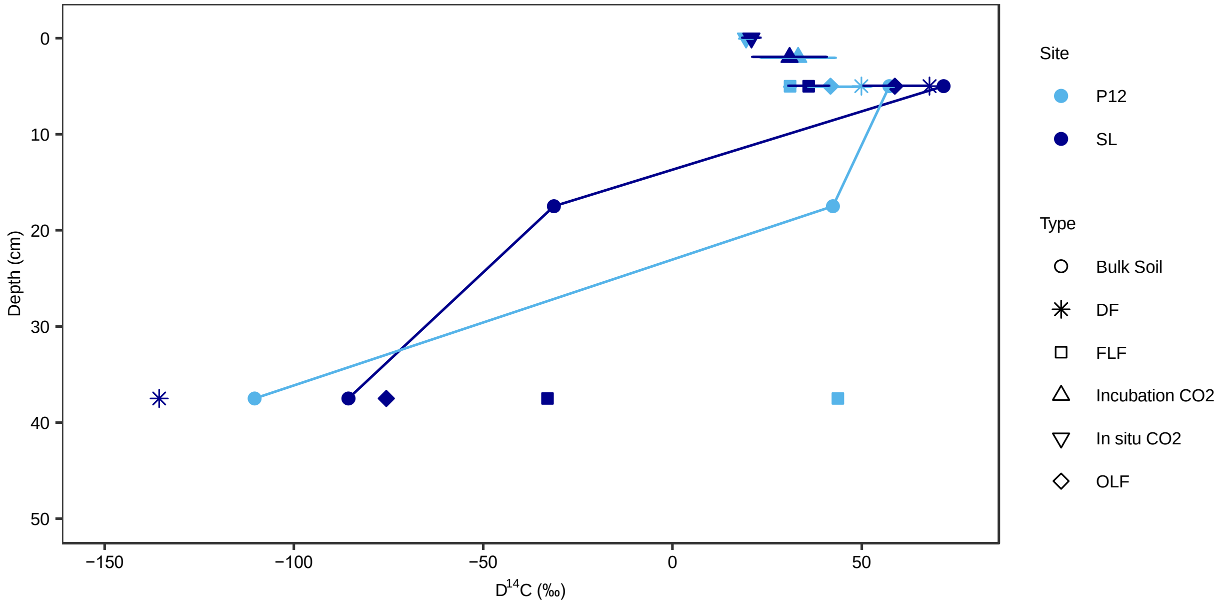

Supplementary Fig. 6: Δ14C values for bulk soil, density fractions CO_2 from laboratory soil incubations, and *in situ* surface soil CO_2 efflux at P12 and SL. *In situ* soil CO_2 efflux $\Delta^{14}C$ values are from this study, are the same as those shown in Fig. 4, and are replotted (for all collar types and seasons) for reference. The figure shows means as symbols with standard errors. n = 3 for bulk soils, incubations, and density fractions from 0–10 cm depth and n = 1 or all sample types at 10–25, and 25–50 cm soil depths. In the 0–10 cm depth, bulk soil and dense fractions have higher $\Delta^{14}C$ values than respired CO_2 ($p < 0.01$). Cordeiro et al., in review observed that deep roots (90–120 cm) at P12 and SL span a wide range of annual to decadal-aged carbon ($\Delta^{14}C$ of 4–86 ‰)[2].

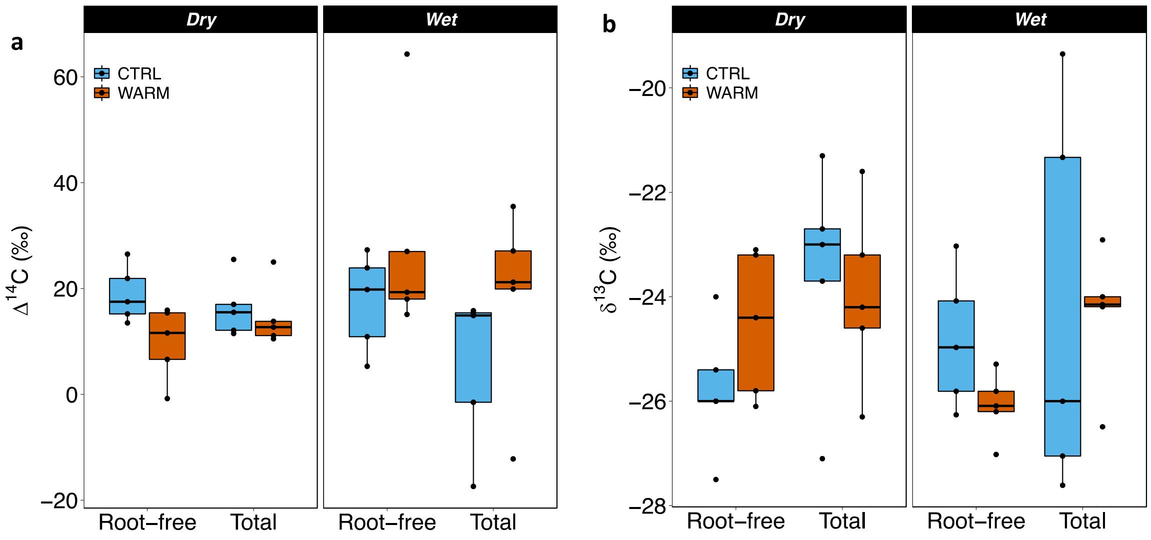

Supplementary Fig. 7: Single time-point isotopic values of respired CO_2 from root exclusion (Root-free) and total soil (Total) respiration collars with and without warming at SWELTR. a. ^{14}C. **b.** ^{13}C. Lines indicate medians, ends of boxes show the upper (Q3) and lower (Q1) quartiles, whiskers indicate minimum and maximum ranges (calculated from quartiles), solid points are individual observations, and n=5. The $\delta^{13}C$ of respired CO_2 averaged -24.7 ± 1.9 ‰ and did not differ with warming ($p = 0.85$) or season ($p = 0.50$) as tested with a three-way repeated measures ANOVA with site, treatment, and season.

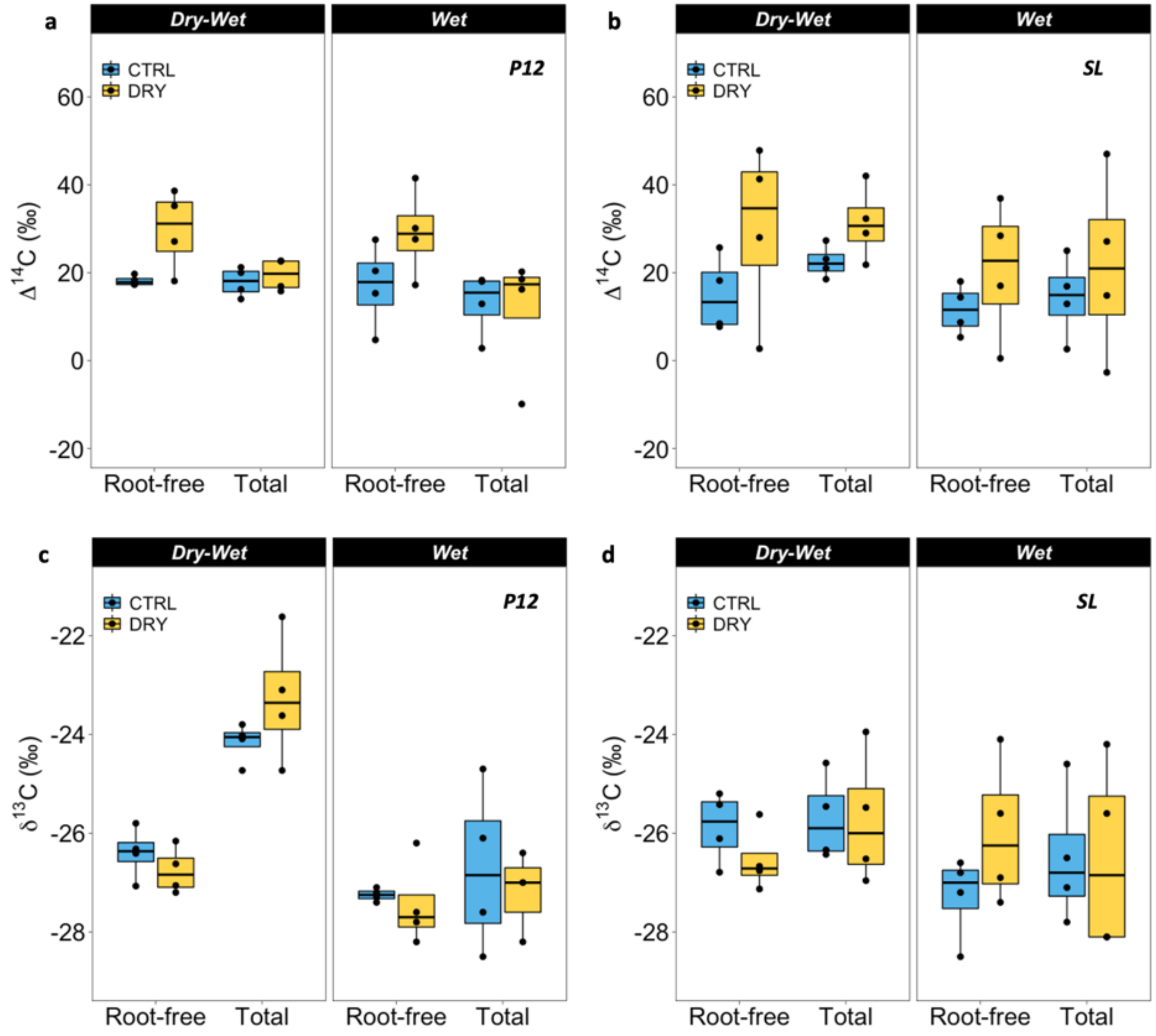

Supplementary Fig. 8: Single time-point isotopic values of respired CO_2 from root exclusion (Root-free) and total soil (Total) respiration collars with and without throughfall exclusion. a. ^{14}C at P12. **b.** ^{14}C at SL**. c.** ^{13}C at P12. **d.** ^{13}C at SL. Lines indicate medians, ends of boxes show the upper (Q3) and lower (Q1) quartiles, whiskers indicate minimum and maximum ranges (calculated from quartiles), solid points are potential outliers, and n=5. The rate of soil respiration was not correlated with its $\Delta^{14}C$ or $\delta^{13}C$ signature, although there was a negative correlation between the $\Delta^{14}C$ and $\delta^{13}C$ of respired CO_2 (ρ = -0.45, n = 40, $p < 0.01$). Pearson correlation analysis was used to assess dependence among soil respiration variables and with soil temperature and moisture using the Hmisc (v. 5.1.1) package[3]. The $\delta^{13}C$ of respired CO_2 averaged did not differ with experimental drying (p = 0.44) as tested with a three-way repeated measures ANOVA with site, treatment, and season.

Supplementary Tables 1–9

Supplementary Table 1. Study Site Characteristics

**Site**	**Site code**	**Experimental Treatment**	**Lat.**	**Long.**	**MAP (mm)**	**Soil order**	**Parent material**
SWELTR	SWT	Warming	9.15	-79.86	2580	Inceptisol	Conglomerate
Plot 12	P12	Drying	9.18	-79.83	2600	Ultisol	Conglomerate
San Lorenzo	SL	Drying	9.28	-79.98	3421	Oxisol	Marine sandstone

Supplementary Table 2. Three-way repeated measures ANOVA results for ^{14}C of CO_2 in soil efflux at SWELTR:

	numDF	denDF	F-value	p-value
(Intercept)	1	16	95.9295	<.0001
Pair.Set	4	12	3.8581	0.0307
Treatment	1	12	1.0085	0.3351
Collar.Type	1	12	2.3241	0.1533
Season	1	16	0.6090	0.4465
Treatment:Collar.Type	1	12	0.4673	0.5072
Treatment:Season	1	16	7.0211	0.0175
Collar.Type:Season	1	16	3.4944	0.0800
Treatment:Collar.Type:Season	1	16	0.2004	0.6604

Supplementary Table 3. Two-way repeated measures ANOVA results for total soil CO_2 efflux at SWELTR:

	numDF	denDF	F-value	p-value
(Intercept)	1	8	134.9171	<.0001
Season	1	8	7.3372	0.0267
Treatment	1	8	6.5162	0.0340
Season:Treatment	1	8	2.6834	0.1400

Supplementary Table 4. Two-way repeated measures ANOVA results for partitioning of total soil CO_2 efflux (portion of total CO_2 efflux from heterotrophs) at SWELTR:

	numDF	denDF	F-value	p-value
(Intercept)	1	8	231.0877	<.0001
Treatment	1	8	0.1068	0.7522
Season	1	8	0.9663	0.3544
Treatment:Season	1	8	0.6212	0.4533

Supplementary Table 5. Four-way repeated measures ANOVA results for ^{14}C of CO_2 in soil efflux at PARCHED:

	numDF	denDF	F-value	p-value
(Intercept)	1	24	141.0051	<.0001
Collar.Type	1	24	0.5410	0.4691
Site	1	24	0.1739	0.6804
Treatment	1	24	5.4977	0.0276
Season	1	24	9.3774	0.0054
Collar.Type:Site	1	24	2.7184	0.1122
Collar.Type:Treatment	1	24	1.3777	0.2520
Site:Treatment	1	24	0.3658	0.5510
Collar.Type:Season	1	24	1.2335	0.2777
Site:Season	1	24	1.1024	0.3042
Treatment:Season	1	24	0.4845	0.4931
Collar.Type:Site:Treatment	1	24	0.3374	0.5667
Collar.Type:Site:Season	1	24	0.1677	0.6858
Collar.Type:Treatment:Season	1	24	0.0005	0.9827
Site:Treatment:Season	1	24	0.0951	0.7604
Collar.Type:Site:Treatment:Season	1	24	0.3037	0.5867

Supplementary Table 6. Three-way ANOVA results for total soil CO_2 efflux at PARCHED:

	numDF	denDF	F-value	p-value
(Intercept)	1	18	92.0939	<.0001
Site	1	6	2.9931	0.1343
Treatment	1	18	6.6954	0.0186
Season	1	18	117.7474	<.0001
Site:Treatment	1	18	1.2614	0.2762
Site:Season	1	18	2.9884	0.1010
Treatment:Season	1	18	4.3767	0.0509
Site:Treatment:Season	1	18	1.1525	0.2972

Supplementary Table 7. Three-way repeated measures ANOVA results for heterotrophic soil CO_2 efflux at PARCHED:

	numDF	denDF	F-value	p-value
(Intercept)	1	12	97.9986	<.0001
Site	1	12	9.9577	0.0083
Treatment	1	12	4.1668	0.0638
Season	1	12	64.0468	<.0001
Site:Treatment	1	12	1.2769	0.2806
Site:Season	1	12	7.0254	0.0212
Treatment:Season	1	12	4.8380	0.0482
Site:Treatment:Season	1	12	0.3708	0.5539

Supplementary Table 8. Three-way repeated measures ANOVA results for root soil CO_2 efflux at PARCHED:

	numDF	denDF	F-value	p-value
(Intercept)	1	12	30.0382	0.0001
Site	1	12	0.0193	0.8918
Treatment	1	12	0.0363	0.8521
Season	1	12	9.3346	0.0100
Site:Treatment	1	12	0.0137	0.9087
Site:Season	1	12	1.2161	0.2918
Treatment:Season	1	12	0.0295	0.8664
Site:Treatment:Season	1	12	0.2625	0.6177

Supplementary Table 9. Three-way repeated measures ANOVA results for partitioning of total soil CO_2 efflux (portion of total CO_2 efflux from heterotrophs) at PARCHED:

	numDF	denDF	F-value	p-value
(Intercept)	1	12	75.8571	<.0001
Site	1	12	0.3475	0.5665
Treatment	1	12	0.1193	0.7358
Season	1	12	8.3573	0.0136
Site:Treatment	1	12	0.6202	0.4462
Site:Season	1	12	6.0753	0.0298
Treatment:Season	1	12	1.4270	0.2553
Site:Treatment:Season	1	12	0.0150	0.9046

Supplementary Methods

Density fractionation was performed as follows. 100 mL of SPT solution added to 20 g of air-dried, 2 mm sieved soil. Samples were gently inverting to wet all soil and allowed to sit overnight before centrifuging at 3500 rpm for 1 h. Floating material (fLF) was aspirated, washed five times with 150 mL of ultrapure water, transferred into pre-weighed dishes, and dried at 55 ºC for 24 h to evaporate standing water followed by 48 h at 110 ºC. 75 mL of SPT was added to the remaining pellet, samples were mixed with a Lightnin LabMaster LB2 mixer at 1400 rpm for 1 min, then sonicated for 1 min at 80% power (max power 500 W) using a Qsonica Q-500 sonicator while sitting on ice, for a total of 192 J/mL. After letting particles settle for 45 min, samples were centrifuged (Avanti J-15R centrifuge with a JS-4.750 swinging bucket rotor, Beckman-Coulter) at 3500 rpm (2850 x g) for 1 h, then allowed to sit overnight. Floating material (oLF) was aspirated then washed and dried in a similar manner to the fLF. The remaining dense fraction (DF) was rinsed four times with 150 mL ultrapure water with 3 mL of a 1 M $CaCl_2$ / 0.1 M HCl solution mixed in for flocculation. Suspended samples were centrifuged at 3500 rpm (2850 x g) for 20 min for the first two rinses, then 4000 rpm (3720 x g) for 1 h for subsequent rinses. After centrifuging, the supernatant was discarded. The DF was then transferred into pre-weighed dishes and dried in a similar manner to the fLF and oLF except that DF samples were dried at 110 ºC for 48 h or until the sample weight stabilized. Once dry, all fractions were weighed and ground for isotopic and chemical analyses.

Supplementary References

1 Hua, Q. *et al.* Atmospheric Radiocarbon for the Period 1950-2019. *Radiocarbon* **64**, 723-745, doi:10.1017/Rdc.2021.95 (2022).

2 Cordeiro, A. L. *et al.* Root Characteristics Vary with Depth across Four Lowland Seasonal Tropical Forests. *Ecosystems* (in review).

3 Hmisc: Harrell Miscellaneous (2019).
