# Supplementary material for: Experimental warming and drying increase older carbon contributions to soil respiration in lowland tropical forests
Source: Nat Commun. 2024 Aug 17;15:7084. doi: 10.1038/s41467-024-51422-6 (PMC11330460; doi:10.1038/s41467-024-51422-6)
Supplement: Supplementary file 3 — Reporting Summary [file 41467_2024_51422_MOESM3_ESM.pdf]

nature portfolio

Corresponding author(s): Karis McFarlane

Last updated by author(s): Jul 16, 2024

Reporting Summary

Nature Portfolio wishes to improve the reproducibility of the work that we publish. This form provides structure for consistency and transparency in reporting. For further information on Nature Portfolio policies, see our Editorial Policies and the Editorial Policy Checklist.

Statistics

For all statistical analyses, confirm that the following items are present in the figure legend, table legend, main text, or Methods section.

n/a	Confirmed	
☐	☒	The exact sample size (*n*) for each experimental group/condition, given as a discrete number and unit of measurement
☐	☒	A statement on whether measurements were taken from distinct samples or whether the same sample was measured repeatedly
☐	☒	The statistical test(s) used AND whether they are one- or two-sided<br>*Only common tests should be described solely by name; describe more complex techniques in the Methods section.*
☐	☒	A description of all covariates tested
☐	☒	A description of any assumptions or corrections, such as tests of normality and adjustment for multiple comparisons
☐	☒	A full description of the statistical parameters including central tendency (e.g. means) or other basic estimates (e.g. regression coefficient) AND variation (e.g. standard deviation) or associated estimates of uncertainty (e.g. confidence intervals)
☐	☒	For null hypothesis testing, the test statistic (e.g. *F*, *t*, *r*) with confidence intervals, effect sizes, degrees of freedom and *P* value noted<br>*Give P values as exact values whenever suitable.*
☒	☐	For Bayesian analysis, information on the choice of priors and Markov chain Monte Carlo settings
☒	☐	For hierarchical and complex designs, identification of the appropriate level for tests and full reporting of outcomes
☐	☒	Estimates of effect sizes (e.g. Cohen's *d*, Pearson's *r*), indicating how they were calculated

Our web collection on statistics for biologists contains articles on many of the points above.

Software and code

Policy information about availability of computer code

Data collection	No software was used for collecting data.
Data analysis	The software R (v. 4.3.2) was used for all statistics with the following data packages: nlme v. 3.1.164, phia v. 0.3.1, lme4 v. 1.1.35.1, Hmisc v. 5.1.1, and lsmeans v. 2.30.0

For manuscripts utilizing custom algorithms or software that are central to the research but not yet described in published literature, software must be made available to editors and reviewers. We strongly encourage code deposition in a community repository (e.g. GitHub). See the Nature Portfolio guidelines for submitting code & software for further information.

Data

Policy information about availability of data

All manuscripts must include a data availability statement. This statement should provide the following information, where applicable:

- Accession codes, unique identifiers, or web links for publicly available datasets
- A description of any restrictions on data availability
- For clinical datasets or third party data, please ensure that the statement adheres to our policy

The data generated and used in this study have been deposited at figshare [https://doi.org/10.6084/m9.figshare.24240211] and at the US Department of Energy's Environmental Systems Science Data Infrastructure for a Virtual Ecosystem (ESS-DIVE) [https://data.ess-dive.lbl.gov/datasets/doi:10.15485/2425968]. The data is publicly available at ESS-DIVE and will be publicly available at figshare upon acceptance of the manuscript.

Research involving human participants, their data, or biological material

Policy information about studies with human participants or human data. See also policy information about sex, gender (identity/presentation), and sexual orientation and race, ethnicity and racism.

Reporting on sex and gender	N/A
Reporting on race, ethnicity, or other socially relevant groupings	N/A
Population characteristics	N/A
Recruitment	N/A
Ethics oversight	N/A

Note that full information on the approval of the study protocol must also be provided in the manuscript.

Field-specific reporting

Please select the one below that is the best fit for your research. If you are not sure, read the appropriate sections before making your selection.

☐ Life sciences ☐ Behavioural & social sciences ☒ Ecological, evolutionary & environmental sciences

For a reference copy of the document with all sections, see nature.com/documents/nr-reporting-summary-flat.pdf

Ecological, evolutionary & environmental sciences study design

All studies must disclose on these points even when the disclosure is negative.

Study description	SWELTR is a soil warming experiment with 5 paired plots (5 non-heated control plots and 5 heated plots) at one site. PARCHED is a throughfall exclusion experiment with 4 paired plots (4 non-exclusion control plots and 4 throughfall exclusion plots) at each site. In this study, we used 2 sites from the PARCHED experiment. Treatments were assigned in the field using paired plots to control for local spatial variability with one plot in each pair randomly selected for treatment vs control.
Research sample	The primary research samples are CO2 collected from soil surface flux chamber headspace within the experimental plots as this study focused on treatment effect on the source of C in soil CO2 emissions. Additional samples to provide context for interpretation include CO2 accumulation rates within the chambers ("soil CO2 flux rate" data) and soil samples collected from within the experimental plots at SWELTR and from outside the experimental plots at the PARCHED sites. Soils were not collected from within the PARCHED plots for this study because the short treatment period was not long enough to alter soil carbon pools. Soils were analyzed, physically fractionated, and incubated in the laboratory to provide additional data reported in this study.
Sampling strategy	The field experimental designs were installed prior to this study. We chose to repeat our sample collection in 2 seasons to increase sample size and assess the impact of seasonality on our results. Additional sampling was not possible for this study under this funding support because the COVID pandemic impacted travel, site access, experimental treatment (loss of power and site maintenance).
Data collection	Data and samples were collected in the field by Karis McFarlane, Daniela Cusack, Lee Dietterich, Alexandra Hedgpeth, and Andrew Nottingham. Air and soil samples were labeled in the field with hand-written field notes describing sampling times and conditions. Maria Jose Montero, Makenna Brown, Biancolini Castro, Lily Colburn, and Korina Valencia provided additional support in data collection for CO2 flux rates, soil temperature, and soil moisture, which were recorded by hand in field notebooks and entered into electronic spreadsheets. Kari Finstad performed laboratory density fractionation and incubations and collected associated data for those samples by hand on paper for density fractionation sample weights that were then entered into electronic spreadsheets. Karis McFarlane, Kari Finstad, and Alexandra Hedpeth prepared radiocarbon samples for analysis and Karis McFarlane collected radiocarbon data. Isotopic and elemental data were collected by the analytical instruments into electronic text files.
Timing and spatial scale	Data were collected from SWELTR in March -April and October of 2019. Data were collected from PARCHED in May and November-December 2019. These sampling campaign periods were selected to target the dry and wet season assessing soil warming effects at SWELTR and to target the dry-to-wet transition season and wet season at PARCHED. The timing of these campaigns was chosen to provide the most contrasting seasons for assessing the effects of warming and drying on the source and rate of soil CO2 efflux.
Data exclusions	No data were excluded from analyses.
Reproducibility	The field experiments used in this study are extensive efforts and very unique to the environmental sciences community. To increase the robustness of our findings we: 1) utilized all field-plot replicates available for the 3 sites (e.g. we did not scale down to save cost and effort); 2) we used two sites to assess the impact of drying in case a single site was not representative of the treatment effects; 3) we repeated our sampling in contrasting seasons to assess the temporal dependency of treatment effects with regards to seasonality in moisture; 4) we collected samples from bulk and exclusion surface flux collars to test whether soil collars would impact our observations (they did not).

Randomization	We relied on the pre-existing field study designs which were informed by previous experimental work. We have found at these sites and other field manipulation experiments that control-experimental plot pairing can be an effective way to address and account for spatial variation in the field across the footprints neccesitated by these experimental treatments (plots are often meters in diameter or a side, such that replicate plots must span upwards of 100's of meters within a single experimental site). We account for plot pairing in our statistical analysis in this study.
Blinding	In the laboratory, samples were assigned blind sampling numbers such that chemical analyses were performed without knowledge of the experimental treatment. Blinding in the field is not possible as experimental treatment equipment are highly visible physical equipment.

Did the study involve field work? ☒ Yes ☐ No

Field work, collection and transport

Field conditions	Samples were collected from lowland tropical rainforest during the dry, dry-to-wet transition, and wet season and field weather conditions were consistent with these targeted sampling seasons.
Location	Samples were collected from SWELTR on Barro Colorado Island (9.15/-79.86), P12 (9.18/-79.83), and San Lorenzo (9.28/-79.98).
Access & import/export	We worked with the Smithsonian Tropical Research Institute (STRI) to ensure that samples were collected and exported in a responsible manner and in accordance with relevant permits and local laws. These sites are active ecological research sites maintained by the Smithsonian Tropical Research Institute. Samples were collected and exported in a responsible manner. Any disturbance associated with accessing field sites and collecting samples was performed consistent with directives from STRI. All experimental work, sample collection, and sample export to the United States was done in compliance with local, national, and international laws and regulations with the necessary research, export, and import permits including:<br><br>Panamanian Ministry of the Environment (Miambiente) export permits for soil samples:<br>No. SEX/PO-7-14,issued 11/9/2017 to Daniela Cusack/Karis McFarlane; No. SEX/O-2-18 issued 2/15/2018 to Ben Turner/Karis McFarlane; No. SEX/O-7-19 issued 7/22/2019 to Ben Turner/Karis McFarlane<br><br>USDA-APHIS Soil Import Permits to Karis McFarlane #P330-18-00326 issued 11/13/2018; P#330-15-00308 issued 11/29/2015<br><br>Smithsonian Tropical Research Institute scientific permits #5671 "Drying Effect on Roots" to Daniela Cusack; #4970 "Soil carbon and climate warming in tropical forests: using experimentation and elevation to reveal responses across space and time" to Andrew Nottingham
Disturbance	These sites are active ecological research sites maintained by the Smithsonian Tropical Research Institute. Any disturbance associated with accessing field sites and collecting samples was performed consistent with directives from STRI.

Reporting for specific materials, systems and methods

We require information from authors about some types of materials, experimental systems and methods used in many studies. Here, indicate whether each material, system or method listed is relevant to your study. If you are not sure if a list item applies to your research, read the appropriate section before selecting a response.

Materials & experimental systems

n/a	Involved in the study
☒	☐ Antibodies
☒	☐ Eukaryotic cell lines
☒	☐ Palaeontology and archaeology
☒	☐ Animals and other organisms
☒	☐ Clinical data
☒	☐ Dual use research of concern
☒	☐ Plants

Methods

n/a	Involved in the study
☒	☐ ChIP-seq
☒	☐ Flow cytometry
☒	☐ MRI-based neuroimaging

Plants

Seed stocks	N/A
Novel plant genotypes	N/A
Authentication	N/A
